# The Complex Dynamics of Decision-Making at the End of Life in the Intensive Care Unit: A Systematic Review of Stakeholders' Views and Influential Factors

**DOI:** 10.7759/cureus.52912

**Published:** 2024-01-25

**Authors:** Spiros Georgakis, Elena Dragioti, Mary Gouva, Georgios Papathanakos, Vasilios Koulouras

**Affiliations:** 1 Faculty of Medicine, School of Health Sciences, University of Ioannina, Ioannina, GRC; 2 Research Laboratory Psychology of Patients, Families & Health Professionals, University of Ioannina, Ioannina, GRC; 3 Intensive Care Unit, University Hospital of Ioannina, Ioannina, GRC

**Keywords:** paternalism, shared decision-making, do-not-resuscitate order, withholding life-sustaining treatment, withdrawing life-sustaining treatment, end-of-life decision-making

## Abstract

A lack of consensus resulting in severe conflicts is often observed between the stakeholders regarding their respective roles in end-of-life (EOL) decision-making in the ICU. Since the burden of these decisions lies upon the individuals, their opinions must be known by medical, judicial, legislative, and governmental authorities. Part of the solution to the issues that arise would be to examine and understand the views of the people in different societies. Hence, in this systematic review, we assessed the attitudes of the physicians, nurses, families, and the general public toward who should be involved in decision-making and influencing factors. Toward this, we searched three electronic databases, i.e., PubMed, CINAHL (Cumulative Index to Nursing & Allied Health), and Embase. A matrix was developed, discussed, accepted, and used for data extraction by two independent investigators. Study quality was evaluated using the Newcastle-Ottawa Scale. Data were extracted by one researcher and double-checked by a second one, and any discrepancies were discussed with a third researcher. The data were analyzed descriptively and synthesized according to the Preferred Reporting Items for Systematic Reviews and Meta-Analyses (PRISMA) guidelines. Thirty-three studies met our inclusion criteria. Most involved healthcare professionals and reported geographic variations in different timeframes. While paternalistic features have been observed, physicians overall showed an inclination toward collaborative decision-making. Correspondingly, the nursing staff, families, and the public are aligned toward patient and relatives’ participation, with nurses expressing their own involvement as well. Six categories of influencing factors were identified, with high-impact factors, including demographics, fear of litigation, and regulation-related ones. Findings delineate three key points. Firstly, overall stakeholders’ perspectives toward EOL decision-making in the ICU seem to be leaning toward a more collaborative decision-making direction. Secondly, to reduce conflicts and reach a consensus, multifaceted efforts are needed by both healthcare professionals and governmental/regulatory authorities. Finally, due to the multifactorial complexity of the subject, directly related to demographic and regulatory factors, these efforts should be more extensively sought at a regional level.

## Introduction and background

Globally, a significant number (16-23%) of intensive care unit (ICU) patients die during their hospital stay, often following a decision to withhold or withdraw life-sustaining treatment [1,2]. The aim of these end-of-life (EOL) practices should primarily be to relieve the patient's suffering when aggressive treatments are deemed futile and death comes as an unavoidable outcome of the disease [3]. However, there is often a lack of agreement among physicians, patients, and families over their respective roles in EOL decision-making [4]. The involvement of nurses has also been a point of interest in various studies [5-17], while the views of the general public have not been comprehensively examined [18].

EOL issues are one of the primary causes of conflicts originating mostly from the lack of knowledge of patients’ wishes [19], comprehension of medical information [20], and discrepancies between the views, values, and beliefs of the stakeholders [21], leading to misperceptions and eventually communication breakdown [19].

Understanding stakeholders' attitudes regarding aspects of EOL and the resulting dynamics can contribute to a viable solution by promoting collaborative decision-making, family meetings, and tailored communication [22,23].

In turn, knowledge of social perspectives can be a useful tool to calibrate communicational and legislative strategies toward high-quality EOL care. This can be achieved by endorsing a cultural movement toward shared decision-making (SDM), especially in light of the recent pandemic events [24]. Legislative and regulatory activity can also provide a solid basis by defining fundamental concepts [14], settling procedures to promote communication between the parties [22], formalizing decision-making mechanisms to identify the wishes of incompetent patients, and adding procedural guidance in the event of a solidified conflict.

EOL practices [1,25-29], attitudes [30-33], and factors [20,34] around decision-making are not novel to relevant literature. However, no study has yet summarized the perspectives of the stakeholders regarding who should be involved in the decision-making process. Hence, herein, we aimed to systematically synthesize the opinions of the physicians, nurses, families, and the public, regarding who should be involved in EOL decision-making in the ICU and influencing factors.

## Review

Study design and registration

This systematic review was conducted following the Preferred Reporting Items for Systematic Reviews and Meta-Analyses (PRISMA, 2020) guidelines [35]. The protocol was registered at the Center for Open Science (OSF: osf.io/8y6ka).

Search strategy and selection criteria

We systematically searched PubMed, CINAHL (Cumulative Index to Nursing & Allied Health), and Embase to identify studies exploring our research subject quantitatively. A broad search strategy was used by combining key terms and MeSH terms related to EOL decisions. No restrictions on demographics or research timeframe were applied. In addition, reference lists of any relevant research studies identified during the screening process were screened for additional relevant articles. The full search terms used for the literature search are noted in Appendix Table A1.

Study selection and eligibility criteria

Two investigators (SG and ED) independently searched titles/abstracts of retrieved references for eligibility, and when a consensus could not be reached, a third author (VK) was consulted. The same procedure was followed at each stage of the search, screening, and selection process. We included all types of studies addressing our population (P), exposure (E), outcome (O), and setting (s) framework (Appendix Table A2).

Studies were considered eligible only if they reflected the attitudes of physicians, nurses, family members, and the general public regarding who should be involved in EOL decision-making for critically ill adult patients (≥18 years of age) admitted in the ICU, reported original research data, and were available in English, full text. We considered EOL decisions as medical decisions to reduce suffering when treatments fail and the patient's quality of life cannot be maintained while recognizing that the possibility of death is an inevitable outcome of the disease’s progression [3,36].

We excluded studies involving children or neonates and non-critical/intensive care settings. We also excluded studies that reported opinions about organ donation and transplantation decisions, euthanasia, or physician-assisted suicide. Organ donation decisions do not have a direct impact on the occurrence of death, while organ transplantation is a life-saving procedure considered a treatment [37]. Euthanasia and physician-assisted suicide should not be confused with EOL practices, as they involve the doctor intentionally aiming for the patient's death, either actively or passively [3,38].

We also categorized the opinions expressed in the eligible studies based on the decision-making models they seemed to reflect. We adopted a broad interpretation of SDM comprising any form of collaborative decision-making where both experts and non-experts discuss or decide together [39].

Studies addressing practices, mere facts, or attitudes about who was actually involved in EOL decisions, e.g., ETHICUS studies [1,26-29,40], were excluded. We also omitted studies examining factors that we considered not to be directly related to opinions about who should participate in these decisions. To limit assumptions and logical leaps, we excluded studies whose data did not clearly capture participants' opinions about who should be included, studies involving children or neonates and non-critical/intensive care settings, and those that reported opinions about organ donation and transplantation decisions or euthanasia or physician-assisted suicide.

Apart from the aforementioned, letters to the editors, opinions, qualitative studies, open-ended surveys, narrative analysis, focus groups, grounded theory, phenomenological and hermeneutic designs, and secondary research studies, such as systematic reviews and overviews of reviews, were excluded.

Study quality assessment

The quality of the included studies was assessed by two independent investigators (SG and ED) using the Newcastle-Ottawa Scale (NOS) [41] indicated in Appendix Table A3.

Data extraction

Data from all full-text papers reviewed were extracted into a predetermined Excel file (Microsoft Corporation, Redmond, WA) by one reviewer (SG) with fields of DOI, author, year, journal, title, study type, population, country, sample details, sample size, setting, year of data collection, aims and objectives, methods, questionnaire type, response rates/respondents, attitudes on who should be involved in EOL decision, factors, main findings, and interesting facts. All data were double-checked by a second reviewer (MG) and any differences in point of view were discussed with a third researcher (VK).

Data synthesis

A narrative synthesis of the included data was performed to provide an overview of the attitudes of the relevant population groups about who should be involved in EOL decision-making and the influencing factors.

Search results

**Figure 1:**
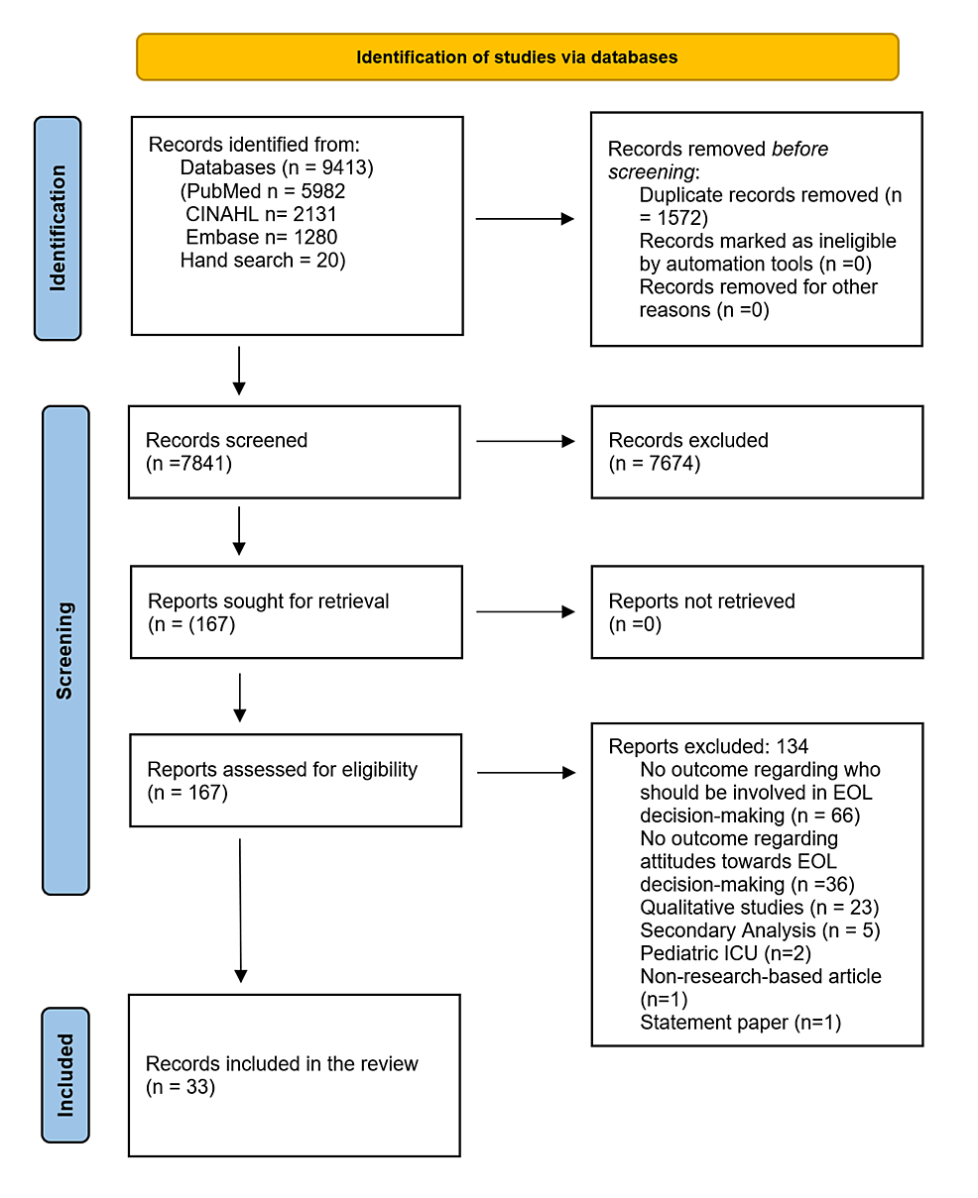
Study selection flowchart. CINAHL: Cumulative Index to Nursing & Allied Health.

From inception to 6th August 2023, a total of 9413 studies were identified through electronic database search: 5982 via PubMed, 2131 via CINAHL, 1280 via Embase, and 20 by hand search (Figure 1). Duplicates were identified and compared based on an exact match of author, year, title, and abstract. After removing 1572 duplicates, 7841 unique records were obtained and screened. We reviewed 167 full-text studies, of which 33 studies were finally included [5-19,42-59].

Characteristics of included studies

The general characteristics of the included studies are described in Table 1. The sample sizes ranged from 41 to 8000 participants (median = 534; interquartile range (IQR) = 186-1200), with most of the surveyed population being physicians. The included publications spanned from 1990 to 2023, with most of them (n = 31) adopting a cross-sectional design. The primary method utilized across studies was the administration of questionnaires. Most of them were conducted at a national level, with the exception of 10 multinational ones [9,12-14,44,45,48,49,57,59]. The individual national studies included four studies from France [5,17,19,50], three studies from the USA [10,16,56], two studies each from Turkey [8,43], China [46,47], and Brazil [42,53], as well as one study each from Israel [51], Croatia [11], South Africa [15], India [58], Italy [54], Portugal [52], Spain [7], Greece [6], Sweden [18], and Canada [55]. Four out of 10 multinational studies were conducted in Europe [9,44,48,49], one in Asia [57], one in Australia and New Zealand [12], one in the Middle East [59], one in both the US and Europe [45], and two on a global scale [13,14].

**Table 1:** General characteristics of the included studies. NR: not reported; EOL: end of life; EuroQODD: Quality of Dying and Death, section of the euroQ2 questionnaire.

First author/year	Country	Study design/level	Period	Population	Sample size (response rate)	Evaluated sample	Assessment method	Attitudes regarding EOL decision-making	Related factors
Giabicani et al., 2023 [19]	France	Cross-sectional, national	June-October 2021	Physicians	186 (86%)	160	In-house questionnaire	Paternalistic	Family-related
Bodas et al., 2023 [51]	Israel	Cross-sectional, national	March 2022	General public	NR	605	Online survey	Shared	Demographics
Špoljar et al., 2022 [11]	Croatia	Cross-sectional, national	October 2018-December 2019	Physicians and nurses	438 (51.5%)	225 total, 116 physicians and 109 nurses	In-house questionnaire	Mixed	Demographics, respondents’ status
Tracy et al., 2022 [12]	Multinational (Australia & New Zealand)	Cross-sectional/multinational	May 28, 2021-July 29, 2021	Physicians and nurses	88 (79.5% completion rate)	70 (29 nurses and 41 physicians)	In-house questionnaire	Shared	NR
Sprung et al., 2021 [13]	Worldwide (32 countries in North America, South America, Eastern Europe, Western Europe, Asia, Australia and South Africa)	Delphi study/multinational	NR	Expert groups of physicians, nurses, non-clinician experts	3049 (45%)	839 physicians, 356 nurses, & 171 non-clinician experts from the general public	In-house questionnaire	Shared	Demographics, existence/knowledge of the existence or absence of relevant legal framework, guidelines and protocols, respondents’ status
Baykara et al., 2020 [43]	Turkey	Cross-sectional/national	NR	Physicians	2,004 (30,5%)	595 (613 questionnaires were retrieved but a total of 595 questionnaires with at least 95% completed answers were included in the analysis)	In-house questionnaire	Mixed	Demographics, existence/knowledge of the existence or absence of relevant legal framework, guidelines, and protocols
Park et al., 2018 [57]	Asia (Japan, China & Korea)	Cross-sectional/multinational	May-December 2012	Physicians	951 (63.6%) total, 345 (56.4%) from China, 265 (70.2%) from Korea, 341 (65.5%) Japan	605 total, 195 from China, 186 from Korea, 224 from Japan	In-house scenario-based questionnaire	Mixed	Demographic factors, fear of litigation, existence/knowledge of the existence or absence of relevant legal framework, guidelines, and protocols
Gerritsen et al., 2018 [44]	Denmark and Netherlands	Cohort/multinational	October 2014-June 2015	Patient families	1,485 (72.5%)	217 out of 1077 responded. Of these, 217 family members of patients who died in the ICU completed the relevant section	Questionnaire (the euroQODD questionnaire, which was distributed as a section of the euroQ2 questionnaire)	Shared	NR
Lomero-Martínez et al., 2018 [7]	Spain	Cross-sectional/national	2013	Physicians and nurses	143 (93%)	133 (70 physicians and 63 nurses)	In-house questionnaire	Paternalistic	Existence/knowledge of the existence or absence of relevant legal framework, guidelines and protocols, patient/family-related factors, respondents’ status
Ntantana et al., 2017 [6]	Greece	Cross-sectional/national	June-December 2015	Physicians and nurses	714 (65.7%)	469 (149 physicians and 320 nurses)	A questionnaire originally developed by Ferrand et al. [5]	Paternalistic	Fear of litigation, existence/knowledge of the existence or absence of relevant legal framework, guidelines and protocols, patient/family-related factors, respondents’ status
Meltzer et al., 2016 [56]	USA	Cross-sectional/national	May-August 2013	Physicians	1200 (15%)	179	Online survey	Paternalistic	Demographic factors
Badır et al., 2016 [8]	Turkey	Cross-sectional/national	March-April 2012	Nurses	866 (72,3%)	602 (626 questionnaires were retrieved. Of these, 24 were incomplete so 602 were evaluated)	A questionnaire originally developed by Latour et al. (2009) [9]	Shared	NR
Metaxa & Lavrentieva, 2015 [45]	USA and Europe	Cross-sectional/multinational	NR	Physicians	150 (27%)	41	In-house questionnaire	Shared	Patient/family-related factors
Langley et al., 2014 [15]	South Africa	Cross-sectional/national	NR	Nurses	149 (67%)	100	A modified version of the questionnaire originally developed by Latour et al. (2009) [9]	Shared	Demographic factors
Sprung et al., 2014 [14]	Worldwide (32 countries in North America, South America, Eastern Europe, Western Europe, Asia, Australia and South Africa)	Delphi study/multinational	NR	Expert groups of physicians, nurses, and non-clinician experts	3049 (45%)	839 physicians, 356 nurses, & 171 non-clinician experts from the general public	Questionnaires	Shared	NR
Ur Rahman et al., 2014 [59]	Multinational (Middle East)	Cross-sectional/multinational	October 2007-January 2008	Physicians	186 (46%)	86	A modified questionnaire originally developed by Vincent [49]	Mixed	NR
Forte et al., 2012 [53]	Brazil	Cross-sectional/national	February-June 2009	Physicians	118 (89%)	105	A questionnaire originally developed by Vincent [49]	Shared (not with nurses)	Demographic factors
Weng et al., 2011 [46]	China	Cross-sectional/national	June-August 2008	Physicians	534 (59%)	315	A questionnaire originally developed by Vincent [49]	Mixed	Fear of litigation, existence/knowledge of the existence or absence of relevant legal framework, guidelines and protocols, patient/family-related factors
Fumis & Deheinzelin, 2010 [42]	Brazil	Cross-sectional/national	NR	Physicians, nurses, and Families	176 (88%) physicians, 215 (94,5%) nurses, and 443 (67,7%) family members	155 physicians, 204 nurses, and 300 family members	A questionnaire originally developed by Sjökvist et al. [18]	Shared	Patients’ state of competence, respondents’ status
Westphal & Mckee, 2009 [16]	USA	Cross-sectional/national	NR	Physicians and nurses	NR	96 total, 53 physicians, and 43 nurses	In-house questionnaire	Shared	Fear of litigation
Latour et al., 2009 [9]	Europe	Cross-sectional/multinational	NR (distributed in November 2005)	Nurses	419 (39,1%)	162 (164 questionnaires were retrieved but 2 of the respondents were outside Europe so they were excluded)	In-house questionnaire	Shared	NR
Barnett & Aurora, 2008 [58]	India	Cross-sectional/national research	November 2002	Physicians	400 (30%)	122	In-house questionnaire	Shared	NR
Azoulay et al., 2004 [17]	France	Prospective/national	The study started on May 1, 2001	ICU staff members (physicians, nurses, assistant nurses) and family members	NR	2,754 ICU staff, 1,473 nurses (54% of ICU staff), 725 assistant nurses (26%), 542 physicians (20%), and 14 physiotherapists (0.5%)/544 family members	Mixed (interview + in-house questionnaire)	Shared on behalf of physicians and nurses/less than half of the families supported their own involvement	ICU staff: family-related factors. Families: patient/family-related factors, respondent status
Giannini et al., 2003 [54]	Italy	Cross-sectional/national	June to July 2001	Physicians	259 (87%)	225	In-house questionnaire	Mixed	Fear of litigation
Ferrand et al., 2003 [5]	France	Cross-sectional/national	July-September 2000	Physicians and nurses	915 (56.9%) physicians and 6,341 (49.8%) nurses	521 physicians and 3,156 nurses	In-house questionnaire	Shared	Fear of litigation, respondents’ status, patient/family-related factors
Azoulay et al., 2003 [50]	France	Cross-sectional/national	February 2002	General public	NR	8000	Interview by telephone/survey questions	Shared	Demographic factors were addressed and were not associated with the attitudes
Cardoso et al., 2003 [52]	Portugal	Cross-sectional/national	October 2001	Physicians	266	175 (66%)	In-house questionnaire	Paternalistic	Demographic factors
Heyland et al., 2003 [55]	Canada	Cross-sectional/national	NR	Families (substitute decision-makers)	1,123 (70.3%)	789	In-house questionnaire	Shared	NR
Yap et al., 2002 [47]	China	Cross-sectional/national	2000	Physicians	95 (68%)	65	A questionnaire originally developed by Vincent [49]	Shared	NR
Sjökvist et al., 1999 [18]	Sweden	Cross-sectional/national	Autumn of 1997	Physicians, nurses, and the general public	121 (88%) physicians, 339 (86%) nurses, and 1,196 (64%) general public	107 physicians, 290 nurses, and 771 general public	In-house questionnaire	Mixed	Patients’ state of competence, respondent status, fear of litigation, existence/knowledge of the existence or absence of relevant legal framework, guidelines, and protocols
Vincent, 1999 [49]	Europe	Cross-sectional/multinational	NR (distributed in April 1996)	Physicians	1,272 (39.6%)	504	In-house questionnaire	Mixed	Demographic factors
Purvis et al., 1998 [10]	USA	Cross-sectional/national	NR (2-week period)	Nurses	57 (91%)	52	In-house questionnaire	Shared	NR
Vincent, 1990 [48]	Europe	Cross-sectional/multinational	NR (distributed in February 1988)	Physicians	590 (41%)	242	Questionnaire (no specific type reported)	Mixed	Demographic factors

Methodological quality of the included studies

The overall summary of the assessment of the included studies according to NOS is described in Appendix Table A3. Overall, 19 studies were assessed as good/high quality and 14 studies were considered of fair quality.

Physicians’ attitudes toward EOL decision-making involvement

Twenty-five studies, 17 national [5-7,11,16-19,42,43,46,47,52-54,56,58] (Table 2) and eight multinational [12-14,45,48,49,57,59] (Table 3) examined physicians’ attitudes. Seven national [5,16,17,42,47,53,58] and four multinational studies [12-14,45] highlighted a collaborative decision-making pattern, while five national studies [6,7,19,52,56] showed a paternalistic one. Notably, five national [11,18,43,46,54] and four multinational studies [48,49,57,59] revealed mixed attitudes.

**Table 2:** Main findings of the studies toward EOL decisions by evaluated population in national studies. N: number of participants per population group; EOL: end of life; LST: life-sustaining treatment; DNR: do not resuscitate; SDM: shared decision-making; DFLST: decisions to forego life-sustaining treatment.

Study	Country	N	Physicians’ attitudes	Nurses’ attitudes	Families’ attitudes	General public’s attitudes
Giabicani et al., 2023 [19]	France	160 physicians	Physicians were not found to support family involvement in medical decisions, also supporting that this would not prevent conflict. Most of them would not apply the decision without taking the family's opposition into account.			
Bodas et al., 2023 [51]	Israel	605 general public				76.5% supported family involvement in EoL decisions.
Špoljar et al., 2022 [11]	Croatia	116 physicians and 109 nurses	78.1% stated that the patient's decision about LST should be respected. Only 26.3% supported collaborative decision-making between themselves and families. Most physicians and nurses are inclined to respect patient's autonomy. However, 55.2% of participants stated that they rarely or very rarely knew the patient’s wishes regarding LST limitation.	80.1% stated that the patient's decision about LST should be respected. If incompetent, 55.8% of them supported physicians and family.		
Meltzer et al., 2016 [56]	USA	179 physicians	Only 32.3% believed that surrogate consent is necessary to discontinue treatment. 56.8% felt they could discontinue over surrogate objection.			
Langley et al., 2014 [15]	South Africa	100 nurses		Most of them stated that their involvement positively influenced job satisfaction. 86% advocated for family involvement. 62% stated that this happened		
Forte et al., 2012 [53]	Brazil	105 physicians	Only 21% would involve nurses. Participants were interested in discussing EOL issues.			
Westphal & Mckee, 2009 [16]	USA	53 physicians and 43 nurses	Inclined toward collaborative decision-making for DNR orders with the nurses. 98% supported patients’ autonomy. 17% claimed that family wishes should be followed over the patient’s living due to fear of litigation.	Inclined toward their involvement in DNR orders with the physicians. 95% supported patients’ autonomy.		
Barnett & Aurora, 2008 [58]	India	122 physicians	73% supported combined EOL decision-making with patients and/or families. The majority of them discussed DNR primarily with families.			
Azoulay et al., 2004 [17]	France	544 family members, 1,473 nurses, 725 assistant nurses, 542 physicians	91% supported family involvement. Only 31% did in practice. More physicians than nurses advocated for family inclusion.	83% of nurses and assistant nurses supported family involvement.	Less than half (47%) supported their own involvement. 15% actually shared decisions.	
Giannini et al., 2003 [54]	Italy	225 physicians	13% would sometimes involve nurses. 56.2% would never involve a competent patient. 70% claimed they rarely/never tried to find out patient’s previously expressed wishes. 58% were not inclined to respect recently expressed patient’s wishes. 58.4% would often/always involve close family in case of an incompetent patient.			
Azoulay et al., 2003 [50]	France	8000 members from the general public				76% wanted family to be included in decision-making.
Cardoso et al., 2003 [52]	Portugal	175 physicians	Regarding all EOL practices, physicians were ambivalent about the involvement of another party. Regarding DNR orders, treatment withholding, and withdrawing, 96%, 95.4%, and 94.9% supported their own involvement. Opinions were split about the involvement of another party (nursing staff/patient/relatives). No specific group was supported by more than half of the respondents. In practice, most decisions were taken solely by physicians.			
Heyland et al., 2003 [55]	Canada	789 families			81.2% of respondents preferred SDM	
Baykara et al., 2020 [43]	Turkey	595 physicians	96.9% believed in their own involvement in end-of-life (EOL) decisions. 69.4% believed in the participation of patients or their legal representatives. However, less than half (41.9%) of ICU physicians supported family involvement in these decisions.			
Ferrand et al., 2003 [5]	France	521 physicians and 3,156 nurses	A majority (80%) believed in collaborative decision-making for decisions to forego life-sustaining treatment (DFLST), but the study did not provide specific data on who should be involved. 75% believed that families should always be informed about DFLST. 61% believed that thorough information should be provided to families. However, despite considering the opinion of nursing staff in treatment decisions (79%), only 36% of physicians considered the presence of nurses necessary during meetings to discuss DFLSTs with families.	91% of nursing staff believed that DFLST should be collaborative, although the specific participants involved were not defined. 56% of nursing staff considered the presence of nurses at meetings to discuss DFLSTs with the family necessary. 75% of nursing staff believed that the family should always be informed, and 69% believed that this should be done fully.		
Yap et al., 2002 [47]	China	65 physicians	The majority of respondents did involve the patient's family (89%) or the patients themselves (52%) in discussions regarding written do-not-resuscitate (DNR) orders. 89% of respondents believed that patients or families should be involved in decisions regarding therapy limitations. However, only 28% of them included ICU nurses in the decision-making process, despite 55% of the physicians recognizing the significance of nurse involvement.			
Weng et al., 2011 [46]	China	315 physicians	Nearly 85% of the physicians believed that decisions regarding the limitation of life-sustaining therapy should include a family representative. 96% believed that discussions about DNR orders should involve the patient or their relatives. Only 19% reported providing complete information, opting instead to adjust the information based on the patient's clinical condition, prognosis, and the recipient's educational level. Only 28% stated that they would disclose all details in the case of an iatrogenic incident.			
Sjökvist et al., 1999 [18]	Sweden	107 physicians, 290 nurses, and 771 members of the general public	In the case of a competent patient, more than half of the physicians were willing to make decisions collaboratively (38% with the patient, 1% with the relatives, and 23% with the patient and the relatives) with a small percentage of them (8%) leaning toward patient’s autonomy. In contrast, when dealing with an incompetent patient scenario, 61% believed they should make the decision alone. Only 36% expressed a preference for involving the family in the decision-making process.	Nurses generally supported shared decision-making regarding the continuation of ventilator treatment in both competent and incompetent patient scenarios. 70% of nurses believed that decisions about the continuation of ventilator treatment should be made jointly by the family and the physician in the incompetent patient scenario. In the competent patient scenario, 57% supported a collaborative decision between the physician, the patient, and/or their family while a smaller percentage (31%) supported patients being the decision-makers alone or together with their families.		Half (50%) of the respondents wanted physicians to be excluded from the decision-making process when the patient is competent. The majority (73%) of respondents advocated for a joint decision made by the family and the physician when the patient is incompetent.
Fumis & Deheinzelin, 2010 [42]	Brazil	155 physicians, 204 nurses, and 300 family members	Physicians were more likely to discuss withdrawal of continued ventilation with the family when a patient is competent compared to when a patient is incompetent (74.8% vs. 60.7%). 71.6% of the physicians believed that such decisions should be made collaboratively by the patient and/or their family together with the physician. In cases of an incompetent patient, 76.8% of the physicians expressed the opinion that decisions regarding ventilator withdrawal should be jointly made by the family and themselves.	75% of nurses believed that discussions about withdrawal of continued ventilation should be held with the family in the competent patient scenario, and 74% in the incompetent patient scenario. 53.4% of nurses believed that decisions about continued ventilator treatment should be shared between the patient and/or family together with the physician in the competent patient scenario, and 78.4% in the incompetent patient scenario.	In the competent patient scenario, 66.3% of family members wanted the decision to be shared between the patient and/or family together with the physician. In the incompetent patient scenario, 78.7% of family members wanted the decision to be shared between the family and the physician.	
Lomero-Martínez et al., 2018 [7]	Spain	70 physicians and 63 nurses	72.9% of the physicians supported joint decision-making between physicians and nurses regarding the application of DFLSTs for the patient. Less than half of them (44.3%) agreed that relatives should actively participate in the decision-making process.	88.9% of nurses believed that decisions to apply limitation of life-sustaining treatment (LLST) should be taken jointly by physicians and nurses. 69.8% of nurses supported family involvement in the decision-making process.		
Ntantana et al., 2017 [6]	Greece	149 physicians and 320 nurses	64.4% of physicians supported the idea that DFLSTs should ideally be taken collaboratively by the nursing staff and physicians. Regarding the information provided to the patient's family, most physicians (66.9%) agreed that the family should be informed. A significant number of physicians (77.8%) also agreed that this information should not be conveyed in great detail due to the relatives' limited understanding of medical specifics.	55.5% of nurses believed that DFLSTs should ideally be taken collaboratively by nursing staff and physicians. 81.1% of nurses believed that the family should be informed about DFLSTs. 73.8% of nurses believed that the information provided should not be done thoroughly due to the relatives' limited understanding of medical details.		
Badır et al., 2016 [8]	Turkey	602 nurses		78.4% of nurses agreed/strongly agreed that patients and/or their families should be fully consulted before a decision to withhold or withdraw treatment. 53.5% of nurses agreed/strongly agreed that involvement in ethical decision-making positively affected their job satisfaction, while 25.2% neither agreed nor disagreed.		
Purvis et al., 1998 [10]	USA	52 nurses		The majority of nurses believed that do-not-resuscitate (DNR) decisions should involve physicians, other team members, mentally competent patients, and their families. 88% of nurses opposed DNR decisions being made exclusively by physicians. 95% of nurses felt that input from mentally competent patients or family members was essential. 56% of nurses believed they should always be involved in DNR decisions, while 21% disagreed.		

**Table 3:** Main findings of the studies toward EOL decisions by evaluated population in multinational studies. N: number of participants per population group; EOL: end of life; SDM: shared decision-making; DNR: do not resuscitate.

Study	Country	N	Physicians’ attitudes	Nurses’ attitudes	Experts’ attitudes (physicians, nurses, and non-clinical experts)	Families’ attitudes
Tracy et al., 2022 [12]	Australia and New Zealand	29 nurses and 41 physicians	More than 95% supported competent patient’s involvement. Almost 85% advocated for the family’s inclusion. More than half of them supported nurse involvement.	All of them supported competent patient’s involvement. Almost all of them supported families and their own involvement. More nurses than physicians supported nurse involvement.		
Sprung et al., 2014 [14]	Worldwide (32 countries in North America, South America, Eastern Europe, Western Europe, Asia, Australia and South Africa)	839 physicians, 356 nurses, and 171 non-clinical experts			Consensus was defined as greater than 80% agreement. A consensus was reached that SDM should be followed with the inclusion of nurses, patients/surrogates/families. Consensus was not reached for withholding or withdrawing treatment only with the consent or agreement of the patient/surrogate/family. A consensus was not reached that it is permissible to withhold or withdraw treatment in situations where an agreement cannot be secured (incompetent patient and no family).	
Park et al., 2018 [57]	Asia (Japan, China, and Korea)	605 physicians (195 from China, 186 from Korea, 224 from Japan)	55.8% and 86.2% of Japanese respondents would (almost always or often) discuss DNR orders with patients and families, respectively. 5.9% and 56.5% of Korean respondents would (almost always or often) discuss DNR orders with patients and families, respectively. 5.1% and 79.5% of Chinese respondents would (almost always or often) discuss DNR orders with patients and families, respectively.			
Sprung et al., 2021 [13]	Worldwide (32 countries in North America, South America, Eastern Europe, Western Europe, Asia, Australia and South Africa)	839 physicians, 356 nurses, and 171 non-clinician experts			Consensus was defined as greater than 80% agreement. A consensus was reached that SDM should be followed with the inclusion of nurses, patients/surrogates/families. A wide majority (more than 3/4) of participants from every region agreed that SDM should be followed. The highest agreement was observed in Asia, North America, South Africa, and South America, and the lowest in Eastern Europe. Consensus was not reached for withholding or withdrawing treatment only with the consent or agreement of the patient/surrogate/family. A consensus was not reached that it is permissible to withhold or withdraw treatment in situations where an agreement cannot be secured (incompetent patient and no family). However, most respondents from all regions (total >70%) agreed. Despite the fact that there was not an equal sample from all regions (ranging from 10 participants from Australia to 688 from Western Europe), this was less common in Asian (withholding and withdrawing) and Eastern European (withdrawing) respondents.	
Ur Rahman et al., 2014 [59]	Middle East	86 physicians	Almost half of the responders (46.5%) wanted physicians to have the ultimate authority in the DNR decision. The majority (62.2%) of the hospitals of the respondents did not have a formal DNR policy.			
Metaxa & Lavrentieva, 2015 [45]	Europe and USA	41 physicians	Less than half (46%) of the participants indicated that they would involve their nursing colleagues in the end-of-life (EOL) process. A small proportion of 27% stated that they would discuss these matters with the team that originally referred the patient. Regarding family involvement, 66% would engage the patient's family in discussions on treatment limitations. More than half (54%) stated that they would explain everything to the family without exception. 46% mentioned that they would adapt the information provided to the family's understanding and/or expectations.			
Vincent., 1990 [48]	Europe	242 physicians	Most of the participants believed that patients and/or their families, along with the entire ICU staff, should be involved in the decision-making process in terminal care. Most participants (66%) supported the idea that DNR orders should be discussed with the patient's family. Interestingly, 68% stated that DNR orders should not be discussed with the patients themselves.			
Vincent., 1999 [49]	Europe	504 physicians	Most physicians (84%) believed that do-not-resuscitate (DNR) orders should be discussed with the patient's family. However, only about half of them (52.7%) supported the idea of discussing DNR orders directly with the patients themselves.			
Latour et al., 2009 [9]	Europe	162 nurses		78.6% of nurses believed that the patient's family should always be consulted in EOL decisions. 71.8% of nurses stated that involvement in EOL decisions positively influenced their job satisfaction.		
Gerritsen et al., 2018 [44]	Denmark and Netherlands	217 patient families				In the Netherlands, 71% of family members preferred shared decision-making, while in Denmark, it was 54%. None of the participants wanted family members to make the decision by themselves. Only a small percentage (5.7%) supported family members making decisions after getting information from doctors. A notable percentage (22.5% in the Netherlands, and 38.4% in Denmark) wanted the physician to make the final decision after discussing it with the family.

Physicians’ Attitudes Highlighting a Collaborative Decision-Making Approach in National Studies

As shown in Tables 2, 4, physicians in the included Chinese [47], American [16], Indian [58], Brazilian [42,53], and French studies [5,17] supported some sort of collaboration with the patients and/or their families. However, in the studies conducted by Ferrand et al. [5] in France and Forte et al. [53] in Brazil, physicians were not keen on nurse involvement. Contrarily, Yap et al. [47] in China as well as Westphal and Mckee [16] in the USA identified more nurse-friendly attitudes.

**Table 4:** Percentage (%) of physicians regarding who should be involved in end-of-life decision-making, as reported in most of the included studies.

First author/year	Physicians’ attitudes toward patients' involvement	Physicians’ attitudes toward families' involvement	Physicians’ attitudes toward patients' and/or families' involvement	Physicians' ultimate authority	Physicians’ attitudes toward their own involvement	Physicians’ attitudes toward nurses' involvement	Physicians’ attitudes toward providing thorough information
Vincent, 1990 [48]	32	66					
Vincent, 1999 [49]	52.7	84					
Sjökvist et al., 1999 (competent patient scenario) [18]	69	24			87		
Sjökvist et al., 1999 (incompetent patient scenario) [18]		36			61		
Yap et al., 2002 [47]	88	92				55	
Ferrand et al., 2003 [5]	80	80				36	61
Fumis & Deheinzelin, 2010 (competent patient scenario) [42]	92.2	92.2			76.8		
Fumis & Deheinzelin, 2010 (incompetent patient scenario) [42]	83.9	83.9			87.1		
Weng et al., 2011 [46]	96	85			75	16	19
Metaxa and Lavrentieva, 2015 [45]		66				46	54
Ntantana et al., 2017 [6]						64.4	22.2
Lomero-Martinez et al., 2018 [7]		44.3				72.9	
Baykara et al., 2020 [43]	69.4	41.9			96.9		
Špoljar et al., 2022 [11]	78	26					
Park et al., 2018 (Japan) [57]	55	86					
Park et al., 2018 (China) [57]	5	79					
Park et al., 2018 (Korea) [57]	6	56					
Meltzer et al., 2016 [56]	68						
Westphal & Mckee, 2009 [16]	98						
Forte et al., 2012 [53]						21	
Barnett & Aurora, 2008 [58]			73				
Azoulay et al., 2004 [17]		91					
Giannini et al., 2003 [54]	44	58					
Cardoso et al., 2003 [52]	38	28			95	29	
Tracy et al., 2022 [12]	95	85					
Ur Rahman et al., 2014 [59]				46			

Paternalistic Attitudes in National Studies

Findings from Portugal [52], Spain [7], Greece [6], France [19], USA [56], and Italy [54] highlight a paternalistic pattern on behalf of the physicians. Interestingly, Lomero-Martínez et al. [7] in Spain identified physicians supporting nurses’ rather than relatives’ involvement, while in Greece, Ntantana et al. [6] observed medical paternalism manifesting in a lack of information provided to the family. However, physicians advocated for nurse involvement. Notably, in the recent study by Giabicani et al. [19] in France, family presence was not thought to reduce potential conflicts, while in Portugal [52], physicians were found hesitant toward the involvement of any other party in decision-making.

Mixed Attitudes in National Studies

Our findings also highlight some mixed attitudes (Tables 1, 2). As reported by Weng et al. [46] in China, despite physicians being in favor of SDM, most of them opted to adjust medical information per occasion. They were also not keen on nurse involvement. Sjökvist et al. [18] revealed Swedish physicians’ preference to collaborate in a competent patient scenario. Reversely, in the case of an incompetent patient, most of them thought themselves to be the sole decision-makers. In Turkey, Baykara et al. [43] reported physicians advocating for patient/legal representative’s involvement, but not for families, while Giannini et al. [54] in Italy found that more than half (58.4%) of the respondents would often/always involve families, but not the patients’ themselves and the nursing staff. Physicians in Croatia, as observed by Špoljar et al. [11], claimed that patient’s autonomy should be respected and advocated for nurses’ involvement. However, only 26.3% of them supported collaboration with the family.

Physicians’ Attitudes in Multinational Studies

As shown in Table 3, multinational studies conducted in Australia and New Zealand by Tracy et al. [12] and in Europe and the USA by Metaxa and Lavrentieva [45], report physicians’ inclination toward patients and families. Nurse involvement was supported by more than half of the physicians in the first study and by less than half (46%) in the second one. In the WELPICUS study [14], more than 80% (consensus) of the experts supported that SDM should be pursued, including patients/surrogates/families and nurses, while more than ¾ of the participants from every region agreed to this [13]. The highest agreement rate was observed in Asia and North America and the lowest in Eastern Europe [13].

Both Vincent’s studies [48,49] highlighted the fact that European physicians’ perspectives differed by country. His first study [48] in the early 90s indicated that most physicians (66%) supported discussing do-not-resuscitate (DNR) orders with the patient's family, but not with the patients themselves (32%). Although no uniformity could be observed, a follow-up study by the same researcher [49] revealed that physicians' attitudes evolved positively toward patient consultation. However, discussion with the patients was supported by less than half of the respondents in most countries.

In their multinational study in Asia, Park et al. [57] identified that most Japanese physicians supported SDM. Interestingly, Korean and Chinese respondents would involve families but not patients (5.9% and 5.1%, respectively).

Finally, almost half (46.5%) of the Middle Eastern participants in the relevant study [42] wanted the ultimate decisional authority regarding DNR orders.

Nurses’ attitudes toward EOL decision-making involvement

As shown in Table 1, 15 studies, 11 national [5-8,10,11,15-18,42] (Table 2) and four multinational [9,12-14] (Table 3), addressed nurses’ attitudes. In all of them, findings consistently revealed nurses’ attitudes supporting SDM (Tables 2, 3). In Spain [7], Turkey [8], Brazil [42], USA [10], South Africa [15], France [5], and Greece [6], they explicitly supported their own involvement (Table 5). These findings were also confirmed by the findings of Latour et al. [9] in Europe and Tracy et al. [12] in Australia and New Zealand. Additionally, nursing staff experts who participated in the WELPICUS study [13,14] reached a consensus with fellow participants supporting SDM, including themselves. Surprisingly, Ntantana et al. [6] observed Greek nurses believing that detailed medical information to the relatives was deemed unnecessary due to limited understanding (Tables 2, 5). This comes in contrast with the findings of Ferrand et al. [5], where nurses supported the opposite.

**Table 5:** Percentage (%) of nurses regarding who should be involved in end-of-life decision-making, as reported in most of the included studies.

First author/year	Nurses’ attitudes toward patient's and/or family's involvement	Nurses’ attitudes toward their own involvement	Nurses’ attitudes toward physician's involvement	Nurses’ attitudes toward providing thorough information
Purvis et al., 1998 [10]	95	56		
Sjökvist et al., 1999 (competent patient scenario) [18]	88		66	
Sjökvist et al., 1999 (incompetent patient scenario) [18]	76		90	
Ferrand et al., 2003 [5]	91	56		69
Latour et al., 2009 [9]	78.6	58.3		
Fumis & Deheinzelin, 2010 (competent patient scenario) [42]	92.6	58.3		
Fumis & Deheinzelin, 2010 (incompetent patient scenario) [42]	92.1	83.9		
Badir et al., 2016 [8]	78.4	53.5		
Ntantana et al., 2017 [6]		55.5	55.5	26.2
Lomero-Martinez et al., 2018 [7]	69.8	88.9	88.9	
Baykara et al., 2020 [43]	69.4	41.9		
Špoljar et al., 2022 [11]	80			
Langley et al., 2014 [15]		86		
Westphal & Mckee, 2009 [16]	95			
Azoulay et al., 2004 [17]	83			
Tracy et al., 2022 [12]	98	93		

Families’ attitudes toward EOL decision-making involvement

Four of the included studies, three national [17,42,55] and one multinational [44], examined families’ attitudes. In brief, findings in Brazil [42] and Canada [55] revealed that, overall, families prefer to have an active role in the decision-making process (Table 2). Similarly, Gerritsen et al. [44] found that most family members in Denmark and the Netherlands also favored SDM but not without physician input. Contrarily, less than half (47%) of the French participants supported their own involvement, as observed by Azoulay et al. [17] (Tables 2, 6).

**Table 6:** Percentage (%) of patients’ family members regarding who should be involved in end-of-life decision-making, as reported in most of the included studies.

First author/year	Families’ attitudes toward physicians' involvement	Families’ attitudes toward their own involvement
Fumis & Deheinzelin, 2010 (competent patient scenario) [42]	70.6	94.3
Fumis & Deheinzelin, 2010 (incompetent patient scenario) [42]	85	92.7
Baykara et al., 2020 [43]	100	62
Azoulay et al., 2004 [17]		47
Heyland et al., 2003 [55]		81

General public's attitudes toward EOL decision-making involvement

Three national studies [18,50,51] assessed the attitudes of the general public (Table 1). Overall, participants from the French [50], Israeli [51], and Swedish [18] public supported family involvement in decision-making (Tables 2, 7). Notably, in a competent patient scenario, half of the respondents (50%) from the Swedish public wanted physicians to be excluded from the decision-making process [18].

**Table 7:** Percentage (%) of participants from the general public regarding who should be involved in end-of-life decision-making, as reported in most of the included studies.

First author/year	General public’s attitudes toward patient's and/or family's involvement	General public’s attitudes toward physician’s involvement
Sjökvist et al., 1999 (competent patient scenario) [18]	96.2	50
Sjökvist et al., 1999 (incompetent patient scenario) [18]	92	73
Bodas et al., 2023 [51]	76	
Azoulay et al., 2003 [50]	76	

Influencing factors

Twenty-four studies [5-7,11,13,15-19,42-46,48-54,56,57] highlighted factors influencing attitudes toward EOL decision-making (Table 1). The results of these studies were grouped into six-factor categories and detailed in Table 8. Briefly, the most common factors among stakeholders were respondents' demographics. Fear of litigation and the absence of legal guidelines and protocols also influenced the views of the physicians and nurses significantly.

**Table 8:** Categories of factors associated with end-of-life decision-making attitudes per study. X = Observed factor influencing end-of-life decision-making attitudes in the relevant study.

First author/year	Fear of litigation	Existence/knowledge of the existence or absence of relevant legal framework, guidelines, and protocols	Patients’ state of competence	Respondents’ status	Patient/family-related factors	Demographics	Study population
Giabicani et al., 2023 [19]					X		Physicians
Bodas et al., 2023 [51]						X (age, marital status, income, religion),	General public
Špoljar et al., 2022 [11]				X		X (sex)	Physicians/nurses
Sprung et al., 2021 [13]	X	X				X (religiosity, medical specialty, country of practice, culture)	Expert groups of physicians, nurses, and non-clinician experts
Baykara et al., 2020 [43]		X				X (medical specialty, age, medical/work experience)	Physicians
Lomero-Martínez et al., 2018 [7]		X		X			Physicians/nurses
Gerritsen et al., 2018 [44]						X (country of practice)	Families
Park et al., 2018 [57]	X	X				X (country of practice, culture)	Physicians
Ntantana et al., 2017 [6]	X	X		X	X		Physicians/nurses
Meltzer et al., 2016 [56]						X (Medical specialty)	Physicians
Metaxa & Lavrentieva, 2015 [45]					X		Physicians
Ur Rahman et al., 2014 [59]						X (country of training, medical/work experience)	Physicians
Langley et al., 2014 [15]						X (medical/work experience)	Nurses
Forte et al., 2012 [53]						X (age, medical specialty, religiosity)	Physicians
Weng et al., 2011 [46]	X	X			X		Physicians
Fumis & Deheinzelin, 2010 [42]			X	X			Physicians/nurses/families
Westphal & Mckee, 2009 [16]	X						Physicians/nurses
Azoulay et al., 2004 [17]				X	X		Physicians/nurses/families
Ferrand et al., 2003 [5]	X			X	X		Physicians/nurses
Giannini et al., 2003 [54]	X						Physicians
Cardoso et al., 2003 [52]						X (medical/work experience, religiosity, sex)	Physicians
Sjökvist et al., 1999 [18]		X	X	X			Physicians/nurses/general public
Vincent, 1999 [49]						X (country of practice, sex, religiosity)	Physicians
Vincent, 1990 [48]						X (age, religion)	Physicians

Fear of Litigation

In Greece [6], France [5], and China [46], fear of litigation was found to influence healthcare professionals' attitudes toward EOL decision-making while also having a direct impact on the quality of the information provided by them to the patients and their relatives, especially in France and Greece. As reported by Westphal and Mckee [16] some physicians in the USA responded that family wishes should be followed even against patient’s wishes due to fear of litigation. In addition, in Italy, Giannini et al. [54] reported that 52% of physicians’ paternalistic attitudes were influenced by fear of legal consequences.

In their multinational study [57] in Asia, Park et al. identify that physicians from China, Korea, and Japan feel exposed to personal legal risk. This was observed mostly in China (49.7%) and Korea (44.1%) where most participants' attitudes endorsed paternalistic features. In the WELPICUS study [13], Sprung et al. stated that fear of litigation may be related to a lack of consensus about withholding or withdrawing agreement without consent in any case, especially in Asia and Eastern Europe.

Existence/Knowledge of the Existence or Absence of Relevant Legal Framework, Guidelines, and Protocols

In Sweden [18], physicians’ paternalistic attitudes (incompetent patient scenario) appear to be correlated to relevant guidelines that were emphasizing on their own decisional authority. On the contrary, according to Baykara et al. [43], up until 2020, the absence of relevant legislation in Turkey placed physicians in difficult situations, obviously influencing their opinions on the subject. This is also supported by the findings of Ntantana et al. [6] in Greece. In China, Weng et al. [46] observed that physicians were generally unaware of the existence of relevant legislation, which was also the case in Spain [7], according to Lomero-Martínez M. et al., for both physicians and nurses, regarding their knowledge of the existence of relevant EOL protocols. Park et al. [57] in their multinational study in Asia observed that most Korean respondents preferred legislative measures to provide guidance regarding EOL issues, while in the WELPICUS study [13], Sprung et al. stated that legal reasons and lack of legal framework may be related to lack of consensus about withholding or withdrawing agreement without consent in any case.

Patients’ State of Competence

In Sweden [18] and Brazil [42], differences were observed among the opinions of the physicians, nurses, patients’ families, and the general public depending on the scenario presented to them (competent vs. incompetent patient). Most worth mentioning disparities were manifested in the attitudes of Swedish physicians who preferred to be the sole decision-makers in case of an incompetent patient but to collaboratively make such decisions in case of a competent one. At the same time, half of the Swedish public excluded physicians’ participation when the patient is competent but advocated for physicians’ involvement in a reverse case.

Perspectives by Respondents’ Status

Lomero-Martínez et al. [7] identified that Spanish nurses were more supportive of collaborative decision-making than physicians. Four other studies (in France [5], Greece [6], Sweden [18], and Brazil [42]) also highlighted disparities regarding attitudes toward EOL decision-making between the examined population categories. A recent study by Špoljar et al. [11] in Croatia highlighted disparities among the perceptions of physicians and nurses toward family involvement with more than half of the latter being in favor of family involvement, a view supported by only 26.3% of the participant physicians. However, physicians were more inclined to respect patients’ wishes than nurses with high school education. Interestingly, another study in France by Azoulay et al. [17] highlighted discrepancies between the opinions of the clinicians and the families. Hence, despite physicians and nurses being in favor of family members’ involvement, this was supported by less than half of the latter.

Patient/Family-Related Factors

In five studies [5,6,19,45,46], patient and family-related attitudes, such as limited understanding of medical details, family involvement would not reduce the risk of conflict, family members do not always decide with the patient’s wishes as an axis, concerns about adding to family’s distress, patient’s clinical condition and prognosis, the recipient’s educational level, and the relatives’ understanding and expectations, were cited as reasons for not disclosing full clinical information. As observed by Azoulay et al. [17], despite less than half of the French family members being in favor of their participation in EOL decision-making processes, those who did, claimed that this should be the case because they considered themselves as best knowledgeable toward patient’s wishes, their opinion was important, and that this would help the ICU staff. Contrarily, those who did not express a desire for participation believed that their presence would not be useful.

Country of Practice

In four multinational studies [13,44,49,57], a country-related lack of uniformity was observed between the participants.

Country of Training

In the Middle East [59], western-trained physicians were more likely to override family and patient decisions about DNR orders.

Culture

In two multinational studies [13,57], culture was observed as an influencing factor. Sprung et al. [13] propose that lack of consensus toward treatment limitation without consent under certain circumstances may be influenced by cultural differences. In Asia, as observed by Park et al. [57], Japanese (29.5%), Chinese (93.8%), and Korean (74.2%) respondents highlighted that not discussing death with a critically ill patient was a part of their culture.

Medical Specialty

In Turkey, Baykara et al. [43] found that compared to physicians whose primary medical specialty was anesthesiology, more internal medicine physicians advocated for family involvement in EOL decisions. Additionally, Meltzer et al. [56] in the USA identified that more physicians (71.4%) in pulmonary/critical care supported that surrogate consent should not be required to discontinue venoarterial extracorporeal membrane oxygenation (VA-ECMO), compared to their colleagues with other medical specialties (general internists (52.8%), cardiologists (52.4%), and others (15.8%)). In the same direction, Forte et al. [53] noted that ICU physicians were more supportive of nurse involvement. In the WELPICUS study [13], it was observed that anesthesiologists were more likely to agree that withholding or withdrawing life-sustaining treatments without the consent of the patient or surrogate should be permissible under certain circumstances.

Sex and Age

Vincent [49] observed that female European physicians in the late 90s discussed DNR orders with patients less frequently than their male colleagues (17% vs. 28%). The same researcher, almost a decade earlier [48], also found that older physicians (>40 years) were more likely to be collaborative in EOL decision-making, which was also confirmed by the work of Baykara et al. [43] in Turkey. Younger age was also associated with attitudes advocating for nurse involvement among physicians in Brazil, as described by Forte et al. [53]. In addition, Bodas et al. [51] reported that Israeli participants from the general public aged more than 70 years agreed to a greater extent (80.4%) toward family involvement than those aged 50 to 59 years (72.6%). Špoljar et al. [11] in Croatia also observed that female physicians and nurses were more inclined than men toward collaborative decision-making with the family. The reverse was observed by Cardoso et al. [52] in Portugal regarding physicians.

Medical/Work Experience

Baykara et al. [43] found that experienced ICU physicians are more supportive of family involvement in EOL decision-making compared to less experienced physicians (less than two years of experience). In the Middle East [59], more experienced physicians were more inclined to override patient/family decisions about DNR orders, while in South Africa [15], nurses with more ICU experience supported family involvement to a greater extent than the rest of their nurse colleagues. In Portugal [52], physicians with >10 years of experience were more supportive of family and nurse involvement.

Religion/Religiosity

In his studies in Europe [48,49], Vincent found that Catholics were less likely to discuss DNR orders with the patients than protestants or agnostics and that almost all religious physicians were more inclined to involve family, nurses, and other ICU staff in EOL decisions. Additionally, Sprung et al. [13] propose that lack of consensus toward treatment limitation without consent under certain circumstances may be influenced by religious differences. Bodas et al. [51] identified that Jew participants from the general population in Israel agreed more than Arabs (77.6% vs. 68.6%, respectively) toward family involvement. Regarding religiosity, Sprung et al. [13] observed that less religious experts were more likely to agree toward life-sustaining treatments without consent under certain conditions. In Brazil, as noted by Forte et al. [53], more religious physicians were more supportive of nurses' involvement. In Portugal, Cardoso et al. [52] found that agnostic/atheist physicians would involve relatives in decision-making more frequently than Catholics.

Marital Status & Income

More divorced than single (78.4% and 66.7%, respectively) and more average income than below average (82.8% and 72.7%, respectively) respondents from the Israeli public would support family involvement in EOL decision-making, as addressed in the relevant study [51].

Discussion

In this systematic review, we aimed to exclusively synthesize and summarize stakeholder’s perspectives regarding who should be involved in EOL decision-making and influential factors.

With one exception [17], the views of the nurses, families, and the public were found to be aligned toward SDM, with the nurses additionally supporting their own involvement.

However, physicians appear to be quite ambivalent in national studies, regardless of the timeframe of the research conduction. Apparently, most of them [3,9,14-16,23,24,27,28,34,40] supported some kind of SDM process. However, paternalistic attitudes were found to correlate with fear of litigation [5,6,16,46,54], absence of relevant framework [6,43,59], or knowledge of its existence and content [7,46], in line with existing literature about EOL practices [1,25,40]. They also manifested mostly indirectly [60] through unwanted family members’ participation [6,7,11,18,43,46,53] or withholding and modifying information [6,46].

The multinational studies [12-14,45,48,49,57,59] demonstrated variations in physician’s attitudes, based on different time periods, healthcare systems, and legal and cultural contexts. A general inclination toward collaborative decision-making was observed. However, paternalistic features did exist in four of these studies [48,49,57,59].

Overall, a societal movement toward SDM was identified. Despite the latter being quite hesitant toward collaboration, to a different extent, nurses and physicians wish to have influence (though not necessarily absolute control) in EOL decisions. There may also be an underlying authority issue on the part of the physicians that nurses wish to balance. From their perspective, families are the most qualified to express their relatives' wishes and should be given the opportunity to be heard.

In line with existing literature about medical practice [1,27], most high-impact factors were observed to be regulation-related and fear of litigation, especially among clinicians [5-7,13,16,18,43,46,54,57], as well as demographics such as medical specialty [43,53,56], sex [11,49,52], work experience [43,52,59], and religion/religiosity [13,19,48,49,52,53]. This highlights the impact that diverse cultural [1,26,27,34] and regulatory frameworks [16,57,61] have on the attitudes of the communities in question.

The COVID-19 period of hospital triage and autonomy limitation [24,62] stressed healthcare and legislative systems globally to the maximum, created vigorous ethical dilemmas, brought prognostic and communicational uncertainty, intensified conflicts, and brought barriers to SDM [24,63]. A recent study in France [19] conducted in the aftermath of the pandemic revealed physicians' hesitancy toward family involvement in light of conflict. Contrarily, the Israeli public [51] supported relatives’ involvement. Under these recent circumstances, stakeholders’ attitudes toward aspects of EOL, especially decision-making, should be more than ever in focus.

Limitations of our review also deserve discussion. Firstly, due to discrepancies between facts or attitudes about what is being done and opinions about what should be done [11,12,52], as well as for needs of coherence and direct relevance with our specific objectives, important studies, like ETHICUS studies [1,20,26,27,34,40,64-66] addressing practices, facts, attitudes toward other aspects of EOL decision-making and relevant factors, were excluded. To limit assumptions, we exclusively included studies that, in our view, illustrated opinions about who should be involved in EOL decision-making clearly and distinctly. However, this assessment was subjective.

Furthermore, the reviewed studies did not exclusively focus on the attitudes regarding decision-making involvement, and the attention given varied. We were also unable to identify any studies meeting our inclusion criteria that were conducted directly considering recent COVID-19 events.

Study categorization was subjective and influenced by the data provided, and response options given by the initial authors. Additionally, the reliance on closed-ended questionnaires in most of them restricts respondent input and may not fully capture the spectrum of their attitudes [5-8,18,42,48,49]. Our findings derived exclusively from the provided data and future studies may introduce new input that could potentially modify them.

The uneven distribution of surveys and the focus on clinicians rather than a representative sample of the general population also restrict the broader applicability of the results. The timeframe of the studies is another important factor to consider, as the evolving legislative and societal landscape can influence the reviewed attitudes. Therefore, our results should be evaluated within the specific social, educational, legal, and scientific context of the time that the relevant research was conducted.

## Conclusions

This systematic review highlighted the diversity and complexity of the stakeholders’ perspectives as well as the dynamics that seem to arise. Overall, a societal movement toward SDM was identified, which aligns with international suggestions, and the findings of relevant literature toward practices. Globally, EOL scenarios occur in a complex context that cannot be uniformly harmonized, comprising personal values and beliefs, as well as diverse cultural and regulatory frameworks. Hence, applicable solutions against conflicts and toward overall high-quality EOL care should be more extensively pursued on a tailored regional level rather than a unified global scale. In turn, multifaceted efforts are needed both in clinical practice (regarding clinicians' handling of EOL situations) and across the governmental/regulatory spectrum to promote the practical implementation of the conceptualization of SDM, emphasize ethical training, and provide guidance. Finally, future studies should include various population categories within specific timeframes by using commonly accepted validated instruments for a more comprehensive illustration of EOL decision-making attitudes.

## Appendices

Table A1

**Table 9:** Full search terms used for literature search. CINAHL: Cumulative Index to Nursing & Allied Health.

PubMed
(end of life decisions OR end-of-life decisions OR end of life decision making OR end-of-life decision making OR life-sustaining therapies OR withholding OR withdrawing OR DNR OR do-not-resuscitate OR de-escalation) AND (critical illness OR critically ill patients OR critical care OR intensive care unit OR ICU OR critical care unit) AND (adult intensive care OR adults)
CINAHL
TX (end of life decisions or end of life discussions or end of life communications) AND TX (intensive care unit or ICU or critical care or critical care unit) AND TX (adults or adult or aged or elderly)
Embase
('treatment withdrawal' OR 'withdrawal, treatment' OR 'withholding treatment' OR 'end of life decision' OR 'end of life decision making' OR 'shared decision' OR 'de-escalation') AND 'intensive care unit' AND ([adult]/lim OR [young adult]/lim OR [middle aged]/lim OR [aged]/lim OR [very elderly]/lim)

Table A2

**Table 10:** PEO(s) framework. PEO(s): population, exposure, outcome, setting.

Population	ICU physicians, nurses, family members of ICU patients, as well as the general public
Exposure	The perspectives and the influencing factors of the opinions of ICU physicians, nurses, family members of ICU patients, and the general public
Outcome	End-of-life decisions, decisions for withholding/withdrawing of life-sustaining measures, do-not-resuscitate orders
Setting	ICU for adult patients (≥18 years of age)

Table A3

**Table 11:** Newcastle-Ottawa Scale scores for the included studies. Each asterisk symbol (*) is equivalent to one score point.

	Selection				Comparability	Outcome		
	Cohort studies							
Study	Representativeness	Selection of the non-exposed cohort	Ascertainment of exposure	Outcome	Comparability	Assessment of outcome	Adequate follow-up time	Adequacy of follow-up cohort
Gerritsen et al., 2018 [44]		*	*	*	**		*	*
Azoulay et al., 2004 [17]	*	*	*	*	**		*	*
Cross-sectional studies								
Study	Representativeness	Sample size	Non-response rate	Ascertainment of the screening/surveillance tool	Comparability	Assessment of the outcome	Statistical test	
Giabicani et al., 2023 [19]	*			*	*	*	*	
Bodas et al., 2023 [51]	*	*		*	*		*	
Špoljar et al., 2022 [11]	*	*		*	*	*	*	
Tracy et al., 2022 [12]	*			*	*	*	*	
Sprung et al., 2021 [13]	*	*		*	*	*	*	
Baykara et al., 2020 [43]	*	*		*	*	*	*	
Park et al., 2018 [57]	*	*		*	*	*	*	
Lomero-Martínez et al., 2018 [7]	*			*	*	*	*	
Ntantana et al., 2017 [6]	*	*		*	*	*	*	
Meltzer et al., 2016 [56]	*				*		*	
Badır et al., 2016 [8]	*	*		*	*	*	*	
Metaxa & Lavrentieva, 2015 [45]	*			*	*	*	*	
Langley et al., 2014 [15]	*			*	*	*		
Sprung et al., 2014 [14]	*	*		*	*	*	*	
Ur Rahman et al., 2014 [59]	*				*	*	*	
Forte et al., 2012 [53]	*			*	*	*	*	
Weng et al., 2011 [46]	*	*		*	*	*	*	
Fumis & Deheinzelin, 2010 [42]	*	*		*	*	*	*	
Westphal & Mckee, 2009 [16]	*				*	*	*	
Latour et al., 2009 [9]	*	*		*	*	*	*	
Barnett & Aurora, 2008 [58]	*	*				*		
Giannini et al., 2003 [54]	*			*	*	*	*	
Ferrand et al., 2003 [5]	*	*		*	*	*	*	
Azoulay et al., 2003 [50]	*	*		*	*	*	*	
Cardoso et al., 2003 [52]	*			*	*	*	*	
Heyland et al., 2003 [55]	*	*		*	*	*	*	
Yap et al., 2002 [47]	*	*		*	*	*	*	
Sjökvist et al., 1999 [18]	*	*		*	*	*	*	
Vincent, 1999 [49]	*	*		*	*	*	*	
Purvis et al., 1998 [10]	*			*	*	*		
Vincent, 1990 [48]	*	*		*	*	*	*	
